# Prognostic role of HER2 amplification based on fluorescence in situ hybridization (FISH) in pancreatic ductal adenocarcinoma (PDAC): a meta-analysis

**DOI:** 10.1186/s12957-016-0792-x

**Published:** 2016-02-20

**Authors:** Xiaoping Li, Hua Zhao, Jianchun Gu, Leizhen Zheng

**Affiliations:** Department of Oncology, Xinhua Hospital, School of Medicine, Shanghai Jiaotong University, Shanghai, 200092 China; Department of Oncology, Baoshan People Hospital, Yunnan, 678000 China

**Keywords:** Pancreatic cancer, HER2, Prognosis, Meta-analysis, FISH

## Abstract

**Background:**

Previous studies have shown that human epidermal growth factor receptor 2 (HER2) may play an important role in the invasion and metastasis of pancreatic cancer, but the relationship between HER2 amplification level and prognosis of pancreatic cancer patients is still controversial. Therefore, we performed a meta-analysis to determine the prognostic significance of HER2 amplification based on fluorescence in situ hybridization (FISH) in patients with pancreatic cancer.

**Methods:**

PubMed, EMBASE, and Web of Science (Jan 2001 to Jun 2015) were searched. Only articles that detect the HER2 amplification by FISH method were included. RevMan 5.3 and STATA version 12 were used to perform this meta-analysis. Pooled calculations were carried out on hazard ratio (HR) and 95 % confidence interval (CI) to assess the risk of disease.

**Results:**

A total of six eligible studies were enrolled in meta-analysis. The univariate analysis results showed that HER2 amplification was not significantly associated with patients’ overall survival (pooled HR, 1.87, 95 % CI, 0.64–5.46, *P* = 0.25), which are maintained in one study of multivariate analysis (HR 0.51, 95 % CI, 0.12–2.14, *P* = 0.358). HER2 amplification also had no correlation with clinicopathological factors such as age, gender, lymph node metastasis, and tumor stage.

**Conclusions:**

Our results showed that HER2 amplification based on FISH may not be a good prognostic factor for survival in patients with pancreatic cancer.

## Background

Pancreatic cancer is the fourth leading cause of cancer death in the USA [1]. With a 5-year survival rate of only 5 %, pancreatic ductal adenocarcinoma (PDAC) is known for having an extremely poor prognosis [2]. The most widely studied prognostic factors are related to pathological characteristics of the pancreatic cancer, including differentiation, tumor stage, and metastasis [3].

The progress we made in the field of gene research can be specifically expressed as a continuous recognition of new biological prognostic factors and provide reference for the development of cancer classification and treatment strategies. The human epidermal growth factor receptor 2 (HER2) protein, the c-erb gene encoding the epithelial growth factor receptor, has been considered to play an important role in tumorigenesis [4]. Early studies showed that HER2 is expressed in many tissues including breast, gastric, lung, and ovarian cancer and may promote cell proliferation and facilitate uncontrolled cell growth [5–7]. HER2 expression is observed in up to 30 % of breast cancer with this receptor being recognized as a poor prognostic marker but also a therapeutic target of a herceptin-based therapy used in both the adjuvant and metastatic setting. The patient’s treatment decisions are increasingly dependent on the accurate detection of HER2 expression.

Amplification of HER2 gene and/or overexpression of HER2 protein have been implicated in the development of pancreatic cancer (PC). The reported rates of HER2 overexpression in patients with PC range from 4 to 50 % [8]. Until now, in patients with pancreatic cancer, whether the amplification of HER2 may be a prognostic factor for survival has been constantly investigated in numerous studies worldwide. However, in contrast to breast cancer, the association between HER2 amplification and the clinicopathological characteristics of PC remains controversial, with some studies suggesting that HER2 amplification is associated with poor prognosis and others showing that it is not an independent prognostic factor of patient outcome [9, 10]. One important factor contributing to this heterogeneity is that the methodologies for HER2 amplification assessment and the criteria for positivity definition varied a lot in different clinical studies.

In 90 % of pancreatic cancers, HER2 protein overexpression is attributable to gene amplification. The expression of HER2 protein was detected by immunohistochemistry (IHC) in routine practice because of simplicity and low costs. IHC is the most widely used method for the assessment of HER2 expression, which scores the membranous immunostaining of the tumor cells ranging from 0 to 3+. Adopting breast cancer scoring criteria for HER2 expression in PC would lead to many false-positive cases. HER2 gene amplification as detected by fluorescence in situ hybridization (FISH) may be a reliable predictor of clinical response to treatment of pancreatic cancer [11, 12]. Therefore, we performed this meta-analysis study to determine the prognostic significance of HER2 amplification based on FISH in patients with pancreatic cancer.

## Methods

### Search strategy and study selection

All studies were searched using the keywords “HER2,” “erbB-2,” or “HER2/neu” and “pancreatic neoplasms,” “survival,” and “prognosis.” In the study, electronic search engines were used for searching: PubMed, EMBASE, and Web of Science (Jan 2001 to Jun 2015). Titles and abstracts were reviewed for appropriate studies which reported the association of HER2 amplification with overall survival (OS) and clinicopathological features in PDAC patients. The full texts of the studies were reviewed to determine if they were relevant to our aim. The reference lists of all articles were also reviewed to identify additional eligible publications. All studies included in this meta-analysis should meet the following criteria: (1) patients with proven diagnosis of pancreatic ductal adenocarcinoma, (2) HER2 amplification evaluation using FISH method, (3) the clinicopathological characteristics between HER2+ and HER2− patients were provided, (4) OS were analyzed according to HER2 status, and (5) the language used in publications was English. The following was the exclusion criteria: (1) the relevant data could not be extracted; (2) the correspondence letters, case reports, and reviews; and (3) duplication of a previous publication. All research results were evaluated independently by two reviewers (Xiaoping Li and Hua Zhao). All possible publications were in the form of a full text to get further assessment to meet the inclusion criteria. Any disagreement was resolved by discussion.

#### Data extraction

Two researchers independently extracted the relevant data from the literature. Data was extracted from the primary publications according to the guidelines of the Cochrane Handbook. Any disagreement was resolved by discussion. Data retrieved from the publications included the following: year of publication, first author’s name, country, sample size, gender, age, disease stage, rate of HER2 amplified, and survival data. The number of HER2 gene amplification was determined by FISH. The criteria used for scoring HER2 by FISH in PDAC were as follows: non-amplified, Her2:cep17 ratio <2; amplified, Her2:cep17 ratio ≥2. Two reviewers (Xiaoping Li and Hua Zhao) assessed the quality of each study included in this meta-analysis using the Critical Appraisal Skills Programme (CASP) assessment tool and the definitions of the seven items for reporting study quality provided by Azami-Aghdash et al. [13]. Most articles compiled in this study had a good quality score. Staging of pancreatic cancer was based on the UICC classification revised in 2012 [14]. The software GetData Graph Digitizer 2.24 (http://getdata-graph-digitizer.com/) was applied to digitize and extract the data from the Kaplan-Meier curve in some articles.

#### Statistical analysis

The software RevMan 5.3 (the Cochrane Collaboration, Copenhagen) and STATA version 12.0 software (Stata Corporation, College Station, TX, USA) were employed. Comparisons of dichotomous measures were performed to calculate odds ratios (ORs) and their 95 % confidence interval (CI). For the pooled analysis of HER2 expression on survival outcome, hazard ratios (HRs) and its 95 % CIs were the recommended summary statistics for meta-analysis of OS. Heterogeneity was assessed using the *I*^2^ test and *Q* test. *I*^2^ > 50 % indicated significant heterogeneity. Meta-analysis of the data using a fixed-effect model was performed when *I*^2^ tests showed no heterogeneity; otherwise, a random-effect model was used. Two or more than two HER2 studies were identified for meta-analysis, while descriptive analysis is performed in the rest of the studies. *P* < 0.05 was considered as statistically significant. Publication bias was assessed by directly assessing the asymmetry of Begg’s funnel plot, and the quantitative evidence was provided by Egger’s test. Sensitivity analysis was also conducted to examine the stability of the results.

## Results

### Study characteristics

**Fig. 1:**
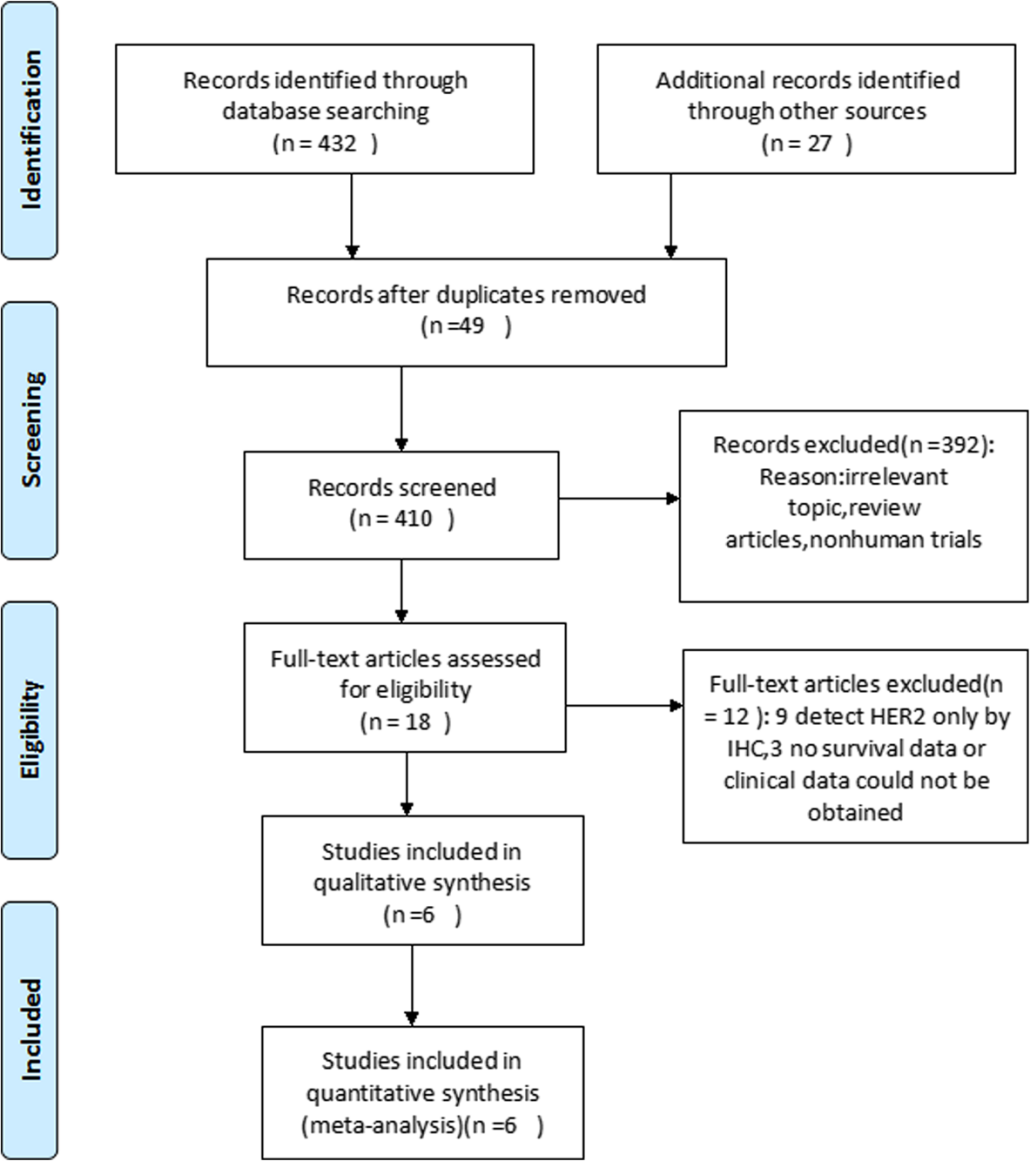
Flow diagram of selection process to identify eligible studies

We identified 459 studies initially eligible for inclusion based on title and abstract screening. After scrutinizing the abstracts, 18 full-text articles were chosen. After further eligibility assessment, 12 articles were further excluded, including two due to survival data not available based on HER2 grouping and ten using only IHC or DISH for HER2 detection (Fig. 1). Finally, we identified six studies from five countries, which used FISH method to detect the HER2 status and provided outcome data stratified by HER2 status. The main characteristics of the six literatures are summarized in Table 1 [9, 10, 15–18]. The total number of pancreatic cancer patients included was 752. Among these studies, one study was performed in China, one in Australia, two in the USA, and two in Germany. All included studies were retrospective cohort studies. According to the CASP quality criteria, most of them were of high quality.

**Table 1:** Main characteristics of all studies included in the meta-analysis

Study	Country	No. patients (M/F)	Age (years)	HER2-positive rate (%)	Follow-up period	Quality score
Dancer 2007 [15]	USA	38 (3/25)	Median 62 (34–82)	10.7	NA	B
Stoecklein 2004 [9]	Germany	50 (28/22)	NA	24	Median 13 months (3–99)	A
Luo 2013 [18]	China	114 (17/97)	NA	14.9	NA	B
Sharif 2008 [16]	USA	63 (NA)	Mean 67 (28–85)	23.8	NA	A
Saxby 2005 [10]	Australia	30 (17/13)	Median 68 (39–82)	3.3	Median 1.7 years	B
Chou 2013 [17]	Australia	469 (241/228)	Median 68 (28–88)	2.1	NA	A

*M/F* male/female, *NA* unknown

#### Correlation between HER2 amplification and clinicopathological parameters

We investigated the association of HER2 amplification status with clinicopathological parameters. The amplified rates of HER2 in the six studies ranged from 2.1 to 23.8 % (Table 1), with a pooled rate of 7.7 % (58/752). The correlation of HER2 amplification with clinicopathological parameters was then studied (Table 2). Three studies reported data on age. Compared with younger patients (≤60 years), pooled data showed that elder patients (>60 years) did not show HER2 amplification (OR 0.71, 95 % CI 0.10–5.21, *P* = 0.74). Furthermore, five studies reported data on gender, two studies reported data on tumor location, six studies reported data on tumor differentiation, four studies reported data on lymph node metastasis, two studies reported data on T stage, and four studies reported data on TNM classification and their relationship with HER2 amplification. When the data was pooled, respectively, there were no significant associations between HER2 amplification and gender (*P* = 0.49), tumor location (*P* = 0.12), differentiation (*P* = 0.55), lymph node metastasis (*P* = 0.76), T stage (*P* = 0.16), and TNM stage (*P* = 0.89) (Table 2).

**Table 2:** Meta-analysis of HER2 amplification in patients with pancreatic cancer

Clinicopathological parameters	No. of studies	Cases	Model	OR	95 % CI	*P* value for OR	*P* value for heterogeneity
Age (≤60/>60)	3	172	R	0.71	0.10–5.21	0.74	0.12
Gender (male/female)	5	691	F	1.25	0.66–2.36	0.49	0.60
Location (head/other)	2	556	R	7.58	0.60–95.35	0.12	0.08
Differentiation (well + moderate/poorly)	6	726	F	0.81	0.42–1.60	0.55	0.33
Lymph node metastasis (yes/no)	4	696	F	1.10	0.59–2.07	0.76	0.96
T stage (T1 + T2/T3 + T4)	2	86	F	0.74	0.34–1.62	0.16	0.45
TNM (I + II/III + IV)	4	645	R	0.87	0.14–5.63	0.89	0.04

*OR* odds ratio, *CI* confidence interval, *F* fixed-effect model, *R* random-effect model

#### Correlation between HER2 amplification and survival outcome

**Fig. 2:**
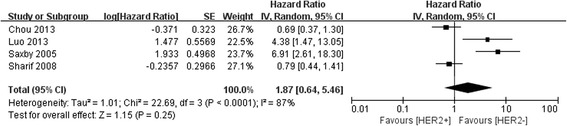
Forest plot of the association between HER2 amplification and OS of pancreatic cancer

We evaluated the correlation between HER2− amplification and survival in a panel of 642 patients from five studies. Meta-analysis on the association of HER2 amplification with OS in patients with PC was carried out. A total of four studies reported OS data in univariate analysis, including one study reporting on OS both in univariate and multivariate analysis. One study reported only RFS data in univariate analysis. Among these pieces of research, two studies found no significant correlation between HER2− amplification and OS, whereas two studies showed that HER2 amplification was a significant association with patients’ prognostic outcomes. Meta-analysis of the four univariate analysis showed that HER2 amplification was not associated with OS (HR 1.87, 95 % CI, 0.64–5.46, *P* = 0.25) (Fig. 2). However, there was significant heterogeneity between the four univariate studies (*P* < 0.0001, *I*^2^ = 87 %). One study reported no significant correlation between HER2− amplification and RFS (HR 0.93, 95 % CI, 0.49–1.78, *P* = 0.823). Compared with tumors without HER2 amplification, those with HER2 amplification were not associated with a statistically significant difference in OS by multivariate analysis (HR 0.51, 95 % CI, 0.12–2.14, *P* = 0.358).

#### Sensitivity analysis and publication bias

**Fig. 3:**
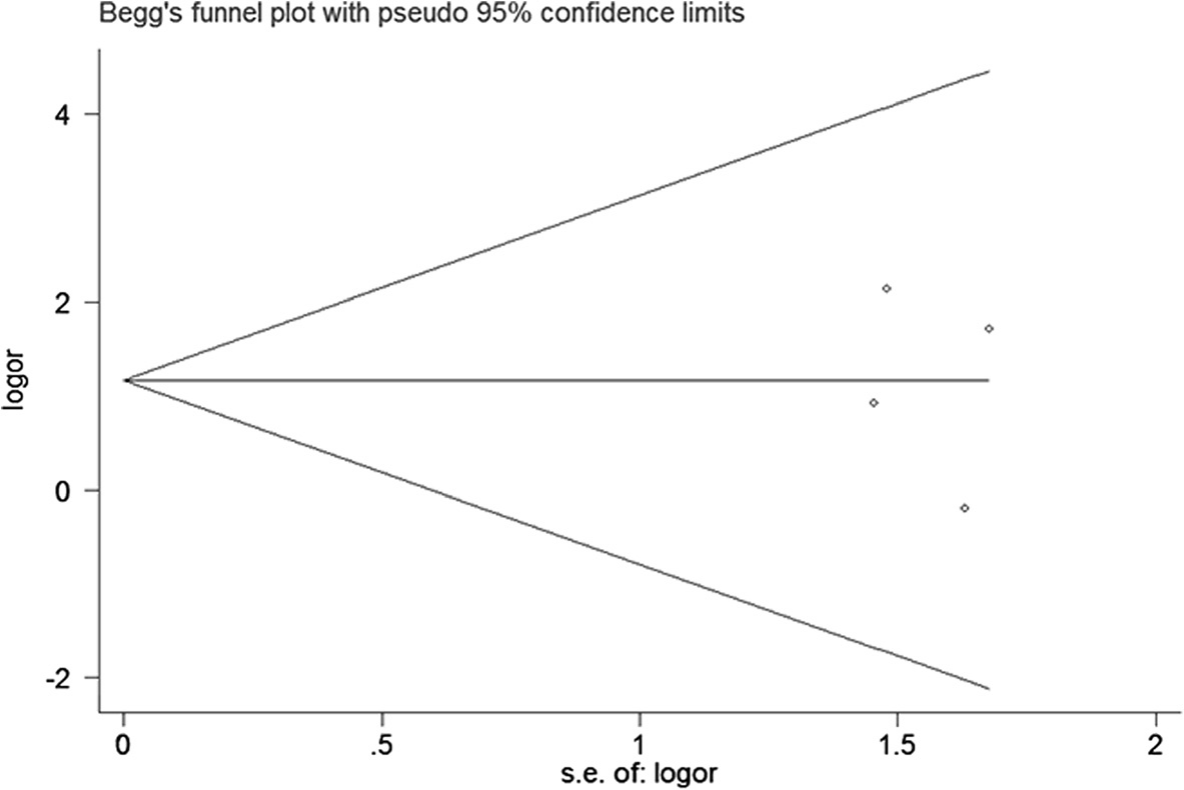
Begg’s funnel plot for visual assessment of publication bias for HER2 amplification and OS of pancreatic cancer

**Fig. 4:**
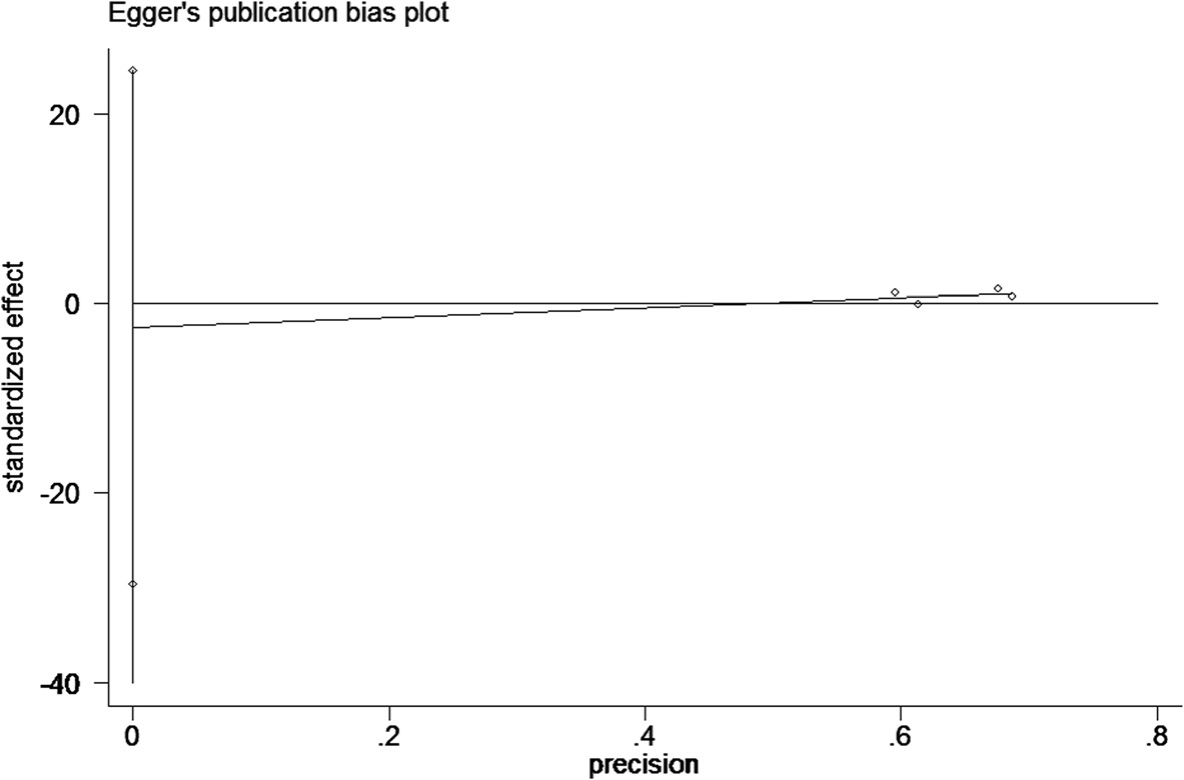
Egger’s publication bias plot showed no publication bias for studies regarding HER2 and OS of pancreatic cancer

Sensitivity analysis to assess the potential impact of each study on pooled HR values was conducted. Results showed that the corresponding combined HR did not change significantly at each step, but the potential heterogeneity was observed again. We further limited the scope of our analysis; quality scores for A were included for meta-analysis. The results showed that HER2 amplification was not significantly associated with OS, and no significant heterogeneity existed (*P* = 0.76). Publication bias was assessed by Begg’s funnel plot and Egger’s test. The results of the funnel plot (Fig. 3) and Egger’s test showed that evidence for symmetry and publication bias did not exist (*P* = 0.723, Fig. 4).

## Discussion

During tumorigenesis, HER2 has been identified as an oncogene involved in the regulation of proliferation, invasion, and apoptosis of tumor cells. A recent report showed that about 42 % of HER2-positive staining and 16 % HER2 gene amplification were observed in pancreatic cancer tissue samples [19]. The expression and amplification of HER2 in various tumor cells not only suggests a poor prognosis but also can be used as an indicator of the corresponding targeted therapy. Preclinical studies showed that the monoclonal antibody trastuzumab can significantly inhibit the growth of pancreatic cancer cell lines and xenograft tumor models [20–22].

Nowadays, IHC is a common method for the determination of HER2 in pancreatic cancer tissues. A major problem with the IHC scoring system for pancreatic cancer is that the morphological and biological characteristics of breast cancer and pancreatic cancer are quiet different. Antigen-retrieval protocols optimized for breast tissue are not necessarily appropriate for pancreas, and some cases did show some membrane labeling in nonmalignant elements, suggesting that the antigen-retrieval process may have been set too high. This may explain that IHC methods have the potential to overestimate HER2-positive status. The other technique is FISH, which is mainly to detect the level of HER2 gene amplification. Precise and accurate detection of HER2 gene expression is crucial in pancreatic cancer to determine the future course of treatment [15].

In this study, we investigated the relationship between the amplification levels of HER2 and clinicopathological factors by meta-analysis. Dysfunction could potentially occur independently at any level in the manufacture of the HER-2 protein such that overexpression exists on the cell surface without genetic amplification. Translational and transcriptional dysfunction has been implicated in other cancer processes to explain this phenomenon. Therefore, such problems will lead to overtreatment or undertreatment by Herceptin. The accuracy of the copy number and the amplification level of HER2 will be of greater clinical significance for Herceptin therapy. This determines whether the technique is trustable or reliable. In pancreatic cancer, Safran et al. [23] addressed this issue in a subset of the IHC group (*n* = 11), finding only three of eight 2+ tumors had gene amplification and none in three 3+ tumors. The finding in this study was in better agreement with FISH than IHC and supports the idea that FISH displays the functional activity of HER-2 gene expression.

In contrast to previous reports, our results showed that HER2 amplification was not related to poor prognosis of pancreatic cancer. There was no statistically significant relationship between HER2 amplification and clinicopathological variables (gender, age, tumor size, grade of differentiation, T stage, and TNM stage). The reasons resulting in this contrary mainly lie in the different criteria for publication selection and enrollment. This study only enrolled the publications that define HER2 status with FISH method, whereas the previous studies used various methods for HER2 detection and scoring.

The significant variability of HER2 amplification definition makes the results incomparable from one study to another. The sample quality and antibody might be important for the difference in FISH positivity. The prognostic significance changes even in the same population if different criteria are used to distinguish HER2+ and HER2− patients. In this study, all included studies define HER2 status with the same criteria (Her2:cep17 ratio ≥2 as the threshold for amplified), which guarantees the credibility of this study. Hofmann et al. [24] proposed a modified HER2 scoring system; the definition for HER2 positivity has changed from previous IHC 2+ or 3+ or HER2 amplification to IHC 3+ or IHC 2+ with HER2 amplification. The principle of this method involves IHC test for HER2 first, furthered by FISH for IHC 2+ patients. The American Society of Clinical Oncology/College of American Pathologists had recommended an updated testing criterion to define HER2-positive status for breast cancer in 2013 [25]. HER2-positive status was defined as follows: there is evidence of protein overexpression (IHC) or gene amplification (HER2 copy number or HER2/CEP17 ratio) by FISH based on counting at least 20 cells within the area. But most reports in our study did not use this criterion; patients received FISH detection directly, so false positive might have existed. Adopting a uniform HER2 amplification definition may be helpful for clinical decision-making in the administration of trastuzumab.

Overexpression of HER2 in breast cancer often indicates a poor prognosis. Different from breast cancer, the role of HER2 as a prognostic factor for pancreatic cancer is still controversial. Saxby et al. [10] suggested that HER2 overexpression was significantly associated with worse prognosis. Chou et al. [17] carried a study included 469 pancreatic cancer patients and got the opposite result. However, in most of the literature, there was little correlation between HER2 amplification and poor prognosis for pancreatic cancer. Our results showed that an HER2-amplification status was not related to the prognosis of pancreatic cancer, and it suggested that the HER2 amplification may not act as a prognostic factor for OS in pancreatic cancer.

The prognosis of PC depended on many clinicopathological factors such as tumor size, location, lymph node metastasis, stage, differentiation degree, etc. Of these factors, tumor size, location, lymph node metastasis, and tumor stage seem to be most important. In Chou’s study [17], they reported that no correlation was found between HER2 amplification and T- or N-factors or TNM stage. Our results also confirmed this. In a series of 30 pancreatic cancers, Saxby et al. [10] reported a general trend of increased HER2 amplification with tumor stage. However, this study included small number patients. Moreover, we also found that there is not much evidence indicating HER2 amplification as a prognostic factor for worse outcome, this also supports the putative role of HER2 in tumor cell aggressiveness but only as a secondary event. We hope to get more information from a larger sample of studies in order to better understand the accuracy of the treatment in the future.

Using anti-HER2 drugs for HER2-positive patients can greatly improve the efficacy of conventional chemotherapy. Harder et al. [26] carried a phase II trial to assess the efficacy and safety of Xeloda and trastuzumab as first-line therapy in patients with HER2-positive metastatic pancreatic cancer. The results showed that the treatment was well-tolerated; however, PFS and OS had no obvious advantages compared with standard chemotherapy. Further research is required to explain the impact of anti-HER2 therapy on the prognosis of metastatic pancreatic cancer.

There are some limitations of this study that should be considered. Firstly, the studies data included in this meta-analysis were all retrospective and nonrandomized ones, so the results may be limited for they are not the highest quality of evidence. Secondly, we cannot rule out that publication bias or heterogeneity between studies may lead to an underestimate of the correlations. Thirdly, we could not get enough data for subgroup analysis such as tumor stage, age, and gender from most of the included studies. Therefore, the prognostic value of HER2 amplification is not clear in subtypes of pancreatic cancer. The software GetData Graph Digitizer was applied to digitize and extract the data from the Kaplan-Meier curve in some articles. This may have led to bias and might not allow a reliable conclusion. Fourthly, the study was restricted to studies published as a full-text article in English, which may lead to bias. Lastly, most reports in this study used FISH detection directly, so we did not know the results of the concordance between IHC and FISH and false positive might have existed.

## Conclusions

In summary, we showed that HER2− amplification based on FISH was not related to clinicopathological features of PC and also not associated with the survival of the patients. As the prognostic value of HER2 for pancreatic cancer is low, anti-HER2 therapy may not be a good choice for pancreatic caner. This can help us to make a decision for optimizing a therapeutic scheme. To further validate our results, larger sample size and well-designed studies are needed to better investigate the prognostic significance of HER2 amplification in pancreatic cancer.
